# Supplementary material for: Blood Serum Cytokines in Patients with Subacute Spinal Cord Injury: A Pilot Study to Search for Biomarkers of Injury Severity
Source: Brain Sci. 2021 Mar 4;11(3):322. doi: 10.3390/brainsci11030322 (PMC8000354; doi:10.3390/brainsci11030322)
Supplement: Supplementary file 1 [file brainsci-11-00322-s001.pdf]

Supplementary Table S1. Cytokine concentrations (ng/mL) in blood serum in patients at 2 weeks post-SCI, including AIS A and B groups and uninjured control subjects.

Markers	Uninjured control	SCI	AIS A	AIS B
**CCL21**	298.26 (297.71)<br>236.11 (80.31–382.39)	414.80 (465.42)<br>356.73 (146.57–465.07)	449.00 (520.65)<br>404.29 (120.17–531.64)	289.39 (84.67)<br>304.15 (250.73–347.31)
**CXCL13**	7.38 (13.40)<br>0.44 (0.19–5.80)	8.10 (17.28)<br>2.25 (1.63–5.33)	5.33 (8.09)<br>2.04 (1.68–4.47)	18.24 (34.34)<br>2.32 (1.51–11.70)
**CCL27**	47.32 (96.73)<br>0.14 (0.10–20.59)	65.12 (275.01)<br>7.13 (3.58–14.15)**	79.03 (310.29)<br>4.75 (3.46–12.50)*	14.11 (7.34)<br>14.20 (9.13–17.38)*
**CXCL5**	119.48 (81.75)<br>109.63 (52.07–139.65)	184.52 (109.71)<br>146.44 (133.27–193.72)*	175.46 (56.60)<br>157.12 (136.83–207.41)*	217.75 (223.29)<br>129.63 (119.32–147.75)
**CCL26**	2.00 (1.46)<br>1.38 (0.92–2.46)	14.86 (6.87)<br>12.29 (10.60–16.84)#	14.40 (5.39)<br>12.42 (10.96–16.51)#	16.55 (11.35)<br>10.41 (9.36–24.20)#
**CCL11**	22.75 (28.30)<br>12.55 (6.92–26.93)	7.57 (3.88)<br>6.08 (4.91–9.42)**	7.44 (3.57)<br>6.29 (4.95–9.22)**	8.02 (5.25)<br>5.15 (4.95–12.15)
**CCL24**	30.08 (51.06)<br>3.97 (1.21–27.26)	19.12 (42.30)<br>7.40 (4.38–16.25)	22.32 (47.41)<br>8.58 (4.32–22.12)	7.39 (3.44)<br>7.05 (4.87–9.79)
**CX3CL1**	61.28 (50.57)<br>41.86 (23.64–99.13)	42.81 (57.80)<br>23.88 (15.54–45.33)	46.27 (64.16)<br>23.88 (16.53–44.45)	30.14 (22.46)<br>20.38 (13.18–44.49)
**CXCL6**	2.61 (2.25)<br>2.07 (0.80–3.11)	9.13 (5.34)<br>7.47 (6.98–9.70)#	8.58 (2.84)<br>7.58 (7.04–9.70)#	11.14 (10.68)<br>7.10 (6.40–8.44)#
**GMCSF**	13.03 (8.07)<br>8.81 (7.62–17.18)	17.36 (9.16)<br>14.16 (11.97–18.38)	16.57 (8.10)<br>14.16 (11.97–18.12)	20.25 (12.81)<br>16.27 (12.52–19.15)
**CXCL1**	26.80 (20.21)<br>18.16 (13.23–40.48)	66.42 (126.72)<br>36.86 (31.02–56.28)**	44.75 (16.17)<br>41.73 (31.88–57.29)*	145.85 (275.63)<br>34.05 (30.29–41.25)*
**CXCL2**	11.04 (14.86)<br>3.62 (2.03–14.68)	8.15 (5.63)<br>6.11 (5.40–8.09)	7.75 (5.83)<br>5.67 (5.40–6.99)	9.62 (4.99)<br>8.09 (7.31–9.64)
**CCL1**	3.25 (2.62)<br>1.90 (1.40–4.96)	9.83 (5.66)<br>8.36 (7.58–9.31)#	8.97 (2.88)<br>8.17 (7.58–8.96)#	12.97 (11.10)<br>8.96 (7.84–9.64)#
**CXCL11**	72.70 (175.50)<br>2.02 (1.21–36.61)	2.02 (1.28)<br>1.66 (1.11–2.34)*	2.04 (1.33)<br>1.66 (1.18–2.23)*	1.94 (1.18)<br>1.65 (1.03–2.32)
**IFNγ**	0.42 (0.20)<br>0.39 (0.24–0.60)	20.71 (7.86)<br>17.92 (16.27–22.99)#	20.47 (6.23)<br>18.46 (16.27–22.02)#	21.61 (13.02)<br>16.27 (14.98–22.38)#
**IL10**	12.40 (7.81)<br>12.13 (6.47–15.23)	6.30 (8.04)<br>3.98 (2.81–5.61)**	5.41 (4.50)<br>4.82 (2.93–5.74)**	9.53 (15.73)<br>3.25 (2.86–3.60)
**IL16**	119.37 (154.81)<br>71.38 (27.64–131.39)	98.90 (169.53)<br>34.00 (28.61–76.84)	83.70 (127.09)<br>34.00 (27.63–80.88)	154.62 (287.44)<br>37.95 (30.08–57.81)
**IL1b**	5.08 (1.67)<br>5.32 (3.77–6.51)	1.53 (1.44)<br>1.02 (0.86–1.45)#	1.43 (1.08)<br>1.03 (0.88–1.54)#	1.89 (2.47)<br>0.85 (0.74–1.19)#
**IL2**	1.64 (0.83)<br>1.79 (0.81–2.39)	4.33 (2.29)<br>3.66 (3.08–4.52)#	4.30 (1.82)<br>3.66 (3.31–4.52)#	4.47 (3.79)<br>3.13 (2.67–3.57)**
**IL4**	1.58 (1.21)<br>0.99 (0.82–2.25)	5.32 (1.87)<br>4.80 (4.29–6.38)#	5.37 (1.80)<br>4.87 (4.43–6.52)#	5.12 (2.30)<br>4.08 (3.58–5.74)#
**IL6**	6.54 (7.19)<br>5.03 (2.45–5.61)	465.37 (2292.97)<br>4.33 (1.68–13.68)	38.02 (112.28)<br>4.33 (1.69–11.78)	2032.33 (4958.69)<br>4.61 (1.68–22.61)
**IL8**	3.92 (3.91)<br>1.72 (0.69–7.43)	25.95 (113.19)<br>0.77 (0.56–1.40)	5.51 (11.73)<br>0.77 (0.55–1.42)	100.89 (245.01)<br>1.02 (0.65–1.29)
**CXCL10**	10.76 (17.69)<br>4.26 (1.79–9.72)	59.30 (160.22)<br>13.36 (5.69–30.63)*	33.92 (64.25)<br>13.36 (5.78–23.13)*	152.35 (328.74)<br>13.70 (5.52–45.89)*
**MCP-1**	12.90 (11.70)<br>8.89 (5.82–16.23)	137.30 (557.66)<br>3.95 (1.74–7.57)	158.23 (627.53)<br>2.72 (1.86–6.60)	60.53 (128.06)<br>4.91 (2.14–23.90)
**MCP-2**	22.55 (21.68)<br>13.55 (5.74–34.23)	10.16 (12.35)<br>5.79 (3.35–12.62)	8.62 (7.37)<br>5.98 (3.81–11.11)	15.81 (23.39)<br>2.79 (2.09–20.54)
**MCP-3**	10.28 (5.16)<br>12.02 (6.15–13.56)	41.46 (16.36)<br>35.93 (32.31–48.86)#	440.95 (13.01)<br>37.12 (32.31–47.79)#	43.34 (27.02)<br>33.53 (26.54–48.11)#
**MCP-4**	13.06 (22.18)<br>0.93 (0.36–16.04)	6.95 (7.25)<br>4.10 (2.67–7.71)	6.39 (6.57)<br>4.10 (2.64–6.65)	9.02 (9.78)<br>5.31 (3.53–10.37)
**CCL22**	10.41 (9.80)<br>7.10 (3.43–14.98)	41.16 (97.74)<br>17.98 (9.97–41.21)**	41.68 (110.51)<br>12.79 (9.92–21.59)*	39.27 (16.98)<br>47.52 (33.02–50.02)**

**MIF**	113.21 (168.20)<br>18.53 (5.88–142.69)	340.15 (1182.84)<br>59.54 (48.36–104.22)	124.79 (247.60)<br>53.08 (44.16–77.47)	1129.79 (2519.01)<br>104.59 (74.06–155.54)*
**MIG**	114.44 (154.44)<br>70.53 (40.30–136.70)	34.36 (31.33)<br>26.35 (16.55–39.52)**	32.98 (19.67)<br>27.23 (18.75–42.68)**	39.45 (60.28)<br>15.63 (10.05–24.82)**
**MIP-1a**	3.45 (3.65)<br>2.19 (1.50–3.95)	2.75 (3.27)<br>1.27 (1.02–2.15)	2.81 (3.26)<br>1.31 (1.02–2.42)	2.52 (3.64)<br>1.08 (0.92–1.25)
**MIP-3a**	0.53 (0.73)<br>0.22 (0.07–0.82)	3.44 (5.89)<br>1.50 (1.20–2.28)#	3.90 (6.58)<br>1.50 (1.33–2.10)#	1.77 (0.98)<br>1.32 (1.09–2.09)**
**MIP1b**	489.82 (963.83)<br>7.35 (3.80–363.86)	1179.69 (3431.55)<br>66.47 (18.33–181.59)	1321.85 (3846.42)<br>28.03 (16.48–99.10)	658.40 (1016.91)<br>156.71 (92.26–691.92)
**MIP3-b**	74.32 (64.38)<br>67.44 (8.54–114.61)	68.46 (130.22)<br>39.57 (16.22–60.43)	51.11 (38.19)<br>46.86 (21.10–62.88)	132.08 (281.54)<br>17.55 (12.33–27.45)
**MPIF-1**	74.37 (148.79)<br>15.55 (6.23–77.32)	391.15 (1848.69)<br>10.94 (7.72–30.63)	486.45 (2085.33)<br>10.94 (7.80–27.85)	41.70 (68.27)<br>10.93 (7.19–30.63)
**CXCL16**	46.38 (59.93)<br>12.91 (0.89–79.78)	70.73 (111.40)<br>24.09 (8.46–96.25)	56.28 (88.66)<br>12.33 (3.64–51.52)	123.71 (172.38)<br>60.31 (20.94–120.67)
**CXCL12**	64.04 (110.42)<br>8.56 (6.09–65.34)	130.58 (274.43)<br>24.99 (9.18–81.99)	102.89 (195.53)<br>23.74 (9.13–78.94)	222.89 (466.87)<br>24.99 (13.05–77.99)
**CCL17**	55.24 (82.44)<br>25.96 (7.71–39.98)	24.02 (17.69)<br>15.73 (11.82–30.03)	26.85 (18.90)<br>22.49 (12.66–33.24)	13.62 (5.06)<br>12.97 (11.34–14.51)
**CCL25**	53.74 (48.19)<br>45.75 (29.97–62.92)	84.10 (59.91)<br>75.08 (43.67–96.85)*	82.23 (46.34)<br>77.57 (48.17–96.85)	90.97 (101.43)<br>46.67 (37.58–77.82)
**TNFa**	2.98 (3.49)<br>1.12 (0.80–3.78)	4.58 (4.70)<br>3.21 (2.60–4.07)*	4.00 (2.05)<br>3.33 (2.64–4.46)*	6.69 (9.75)<br>2.76 (2.51–3.13)

*Padj < 0.05, **Padj < 0.01 and #Padj < 0.0001 comparing to uninjured control subjects.
